# Longitudinal changes in young children’s 0–100 to 0–1000 number-line error signatures

**DOI:** 10.3389/fpsyg.2015.00647

**Published:** 2015-05-15

**Authors:** Robert A. Reeve, Jacob M. Paul, Brian Butterworth

**Affiliations:** ^1^Melbourne School of Psychological Sciences, University of Melbourne, Melbourne, VIC, Australia; ^2^Institute of Cognitive Neuroscience, University College London, London, UK

**Keywords:** number line error signatures, predicting math ability, longitudinal analysis, latent difference scores, stability and change in development

## Abstract

We use a latent difference score (LDS) model to examine changes in young children’s number-line (NL) error signatures (errors marking numbers on a NL) over 18 months. A LDS model (1) overcomes some of the inference limitations of analytic models used in previous research, and in particular (2) provides a more reliable test of hypotheses about the meaning and significance of changes in NL error signatures over time and task. The NL error signatures of 217 6-year-olds’ (on test occasion one) were assessed three times over 18 months, along with their math ability on two occasions. On the first occasion (T1) children completed a 0–100 NL task; on the second (T2) a 0–100 NL and a 0–1000 NL task; on the third (T3) occasion a 0–1000 NL task. On the third and fourth occasions (T3 and T4), children completed mental calculation tasks. Although NL error signatures changed over time, these were predictable from other NL task error signatures, and predicted calculation accuracy at T3, as well as changes in calculation between T3 and T4. Multiple indirect effects (change parameters) showed that associations between initial NL error signatures (0–100 NL) and later mental calculation ability were mediated by error signatures on the 0–1000 NL task. The pattern of findings from the LDS model highlight the value of identifying direct and indirect effects in characterizing changing relationships in cognitive representations over task and time. Substantively, they support the claim that children’s NL error signatures generalize over task and time and thus can be used to predict math ability.

## Introduction

The relationship between age-related changes in number-line (NL) error signatures (deviation errors in marking the location of specified numbers on a horizontal number line—e.g., “67” on a 30 cm 0–100 NL) and math ability have led some to claim that NL signatures are markers of math competence (Siegler and Booth, 2004; Siegler and Ramani, 2009; Sasanguie et al., 2013). Others argue this causal inference is unwarranted since the relationship is merely a correlation between two changing measures, often based on cross-sectional age data. We suggest some insight into the diagnostic relevance of NL error signatures could be gained by examining the stability and/or change in and across NL tasks over time. If NL error signatures remain relatively stable over time and task, it could be interpreted as continuity in NL representations, and support for the claim that NL error signatures are markers of math competence. However, if NL error signatures vary widely across time and task, it would argue against a stable representation, and instead support the claim that NL abilities likely reflect educational experiences.

Determining the stability (or otherwise) of NL error signatures across time and task is more methodologically challenging than it might at first seem. We identified four studies that investigated stability and/or change in NL error signatures over time (Landerl, 2013; LeFevre et al., 2013; Muldoon et al., 2013; Praet and Desoete, 2014), each of which used different analytic models, and which are subject to different interpretive limitations. We use a latent difference score (LDS) model to overcome these limitations and to examine the change and/or stability in 6-year-olds’ 0–100 NL and 0–1000 NL error signatures over a 2 year period.

Decreases in the magnitude of NL errors are correlated with improvements in age-related math abilities (Siegler and Booth, 2004; Booth and Siegler, 2006, 2008; Laski and Siegler, 2007; Schneider et al., 2009; Berteletti et al., 2010; Thompson and Siegler, 2010; Fischer et al., 2011; Ashcraft and Moore, 2012; Sasanguie et al., 2012a,b, 2013; Jordan et al., 2013). At least three hypotheses have been proposed for the correlation. First, something akin to a mental number line (MNL; a subjective scale of numerical magnitudes) is thought to underlie NL estimation abilities (Dehaene and Cohen, 1995; Dehaene, 2001; Siegler and Opfer, 2003; Gilmore et al., 2007). A reduction in NL estimation errors with age is attributed to the fine tuning of a pre-existing magnitude representation system (Siegler and Opfer, 2003; Siegler and Booth, 2004; Booth and Siegler, 2006, 2008; Opfer and Siegler, 2007; Berteletti et al., 2010; Slusser et al., 2012; Kolkman et al., 2013). Second and a related hypothesis is the use of linear representations in formal instruction modifies number-space mapping ability, which in turn supports linear NL representations (Berteletti et al., 2010). Gunderson and colleagues provide longitudinal evidence for a link between early spatial skills, NL acuity and later math abilities. Specifically, the relationship between early spatial skills at age five (e.g., proficiency with mental rotation and translation of shapes) and math ability at age eight (e.g., approximate symbolic calculation) was completely mediated by the linearity of children’s NL estimation responses at age six (Gunderson et al., 2012). Third, improvement in NL estimation abilities simply reflects experiences with NLs in educational settings (Huber et al., 2014). The difficulty with each of these hypotheses is they can only be evaluated using longitudinal research designs.

We could only locate four studies that investigated stability and/or change in NL error signatures over time (Landerl, 2013; LeFevre et al., 2013; Muldoon et al., 2013; Praet and Desoete, 2014). Among other differences, these studies used different analytic models to test claims about the nature of NL error signatures over time, each of which has different limitations on plausible inferences. For instance, change parameters were not explicitly specified in two of the four studies (Landerl, 2013; LeFevre et al., 2013), and latent growth models were fit to a restricted range of change/growth patterns in one study (Muldoon et al., 2013). And assessing NL estimation in a single number range (e.g., 0–100 NLs: LeFevre et al., 2013; Praet and Desoete, 2014) limits conclusions that can be drawn about the generality of NL estimation abilities. When different NL ranges were used (Landerl, 2013; Muldoon et al., 2013), analysis of between-task effects were limited.

In Landerl’s (2013) research, NL estimation abilities were assessed on both 0–100 NL and the 0–1000 NL tasks on five occasions over 2 years (from Grade 2 to 4). The aim was to compare standard regression equations and repeated mean effects that examined changes over time to identify differences in NL estimation between dyscalculic and non-dyscalculic children. Dyscalculic children showed similar patterns of changes in NL estimation abilities over time to non-dyscalculic children (as indicated by decreases in regression slopes). However, the dyscalculic children were consistently less accurate in estimating the position of numbers on NLs (indicated by mean estimation error). The fact that the error signatures showed similar slope patterns for the dyscalculic and non-dyscalculic children on the 0–100 NL and 0–1000 NL was interpreted as showing NL error signatures generalize across different number ranges.

One issue with Landerl’s approach is standard regression models limit inferences that can be drawn about changes to NL error signatures. When linear regression slopes are fit to these data, change is conceptualized as differences in estimation accuracy over time at a constant rate. It is possible that error signatures differ initially and change at different rates; insofar as this is correct it would suggest it is important to take into account the magnitude of initial and subsequent error signatures as well as the rates of change across time.

LeFevre et al. (2013) examined 8-year-olds’ performance on a 0–1000 NL on two occasions. A cross-lagged panel model (CLPM) examined the relationship between differences in NL error signatures, spatial ability and math ability. Results suggested that NL error signatures were correlated across test occasions; however, the authors acknowledge that the direction of influence between NL estimation and math ability was difficult to determine because the two measures appear to affect each other (i.e., were correlated).

Although CLPM potentially provide information about the direction of mutual influence, they do not provide information about change/growth per se. In particular, autoregressive parameters allow an examination of the relative stability of relevant parameters (i.e., how well does prior performance predict current performance). It is important to focus on change as well as relative stability in NL error signatures over time, and the relationship between them and math problem solving.

Muldoon et al. (2013) examined the relationships between NL estimation errors on 0–10, 0–20, and 0–100 NLs in 5-year-olds on four occasions (as well as general math and counting abilities) using latent growth curve modeling. Stepwise linear regressions showed that the linearity of NL estimation error signatures did not predict math ability when counting ability was taken into account (counting ability was used as a proxy for number knowledge). Latent growth curve modeling was subsequently used to determine whether the rate of change in NL error signatures was related to the rate of math ability change (a standardized measure). They found that collinearity between the NL and the math latent growth parameters of NL error impeded model convergence; that is, the high correlation between measures was problematic for the model. The only model to converge suggested that math ability on the first test occasion predicted linearity of NL estimations on the 0–20 NL task on the same occasion, irrespective of the growth parameters. Although most children showed a trend toward linear NL estimation across time, the changes were not captured by their latent growth models.

Although Muldoon et al.’s (2013) analytic approach is an attempt to identify the significance of change parameters associated with NL error signatures directly, the restricted time period of the study may have limited the ability to identify a change model. Moreover, the restriction associated with latent growth curve modeling per se may have also affected outcomes. Latent growth curve analysis requires changes to occur in a systematic manner (i.e., linear, quadratic) for convergence and model fit. Of course there is no a priori guarantee that these conditions will be met for any set of changing relationships.

Praet and Desoete (2014) examined how notation format, intelligence and language skills influenced 0–100 NL estimation at five time points from Kindergarten to Grade 2. Three formats were used to present NL targets: Arabic numerals, spoken/written number words, and dot patterns. Latent growth curve models revealed significant variability in NL estimation accuracy at Kindergarten and that accuracy increased from T1 to T5 with little variability between children for both Arabic numerals and number words. NL estimation accuracy with dot patterns showed similar significant initial variability but also showed significant variability in change of accuracy between children from T1 to T5. Intelligence measured at Kindergarten was a significant covariate predicting both initial percentage absolute error (PAE) in NL estimation, as well as a decrease in PAE from Kindergarten to Grade 2. Language skills measured at Kindergarten also predicted initial variability in PAE in NL estimation but not change over time. However, similar to Muldoon et al. (2013), latent growth curves were specified to change as a linear slope (constant rate) across all time points and only a single NL range was used (0–100 NL).

The different analytic models allow different plausible inferences to be made about NL error signatures. Compared to linear regression analyses, CLPM, LeFevre et al. (2013) provide a characterization of the stability of NL error patterns over time. A useful feature of CLPM is longitudinal associations between a measure and itself at a later time point are taken into account by specifying autoregressive effects (i.e., influence of prior performance on current performance). Autoregressive effects may be useful for examining the persistence of NL error signatures across time (and possibly across task) and whether current performance is best understood in the context of previous performance. Linear regression models do not allow for a characterization of these effects.

Nevertheless, CLPMs do not take into account the effects of prior changes on performance (since the focus is on stability or otherwise of measures over time). There are two difficulties associated with an inability to specify change per se as a parameter in CLPM. First, predictions may over- or under-estimate “direct” (e.g., the influence of “x” on “y”) and “indirect” (e.g., “x” influences “y” though its impact on “z”) effects and their interrelationships are unlikely to be static across time. Second, the interpretation of change itself is not straightforward in CLPMs, given that measures of current performance are likely to be conflated with the accumulated effects of prior changes, both within and across test measures. The aim of the present study is to overcome these limitations using a latent different score model (LDS).

Three issues currently limit the value of NL estimation as an index of later math difficulties: (1) 0–100 NL and 0–1000 NL tasks are rarely assessed together to determine whether children show similar learning across these different number ranges, (2) NL estimation abilities and math problem solving are often assessed at a single time point, limiting conclusions regarding their relationship over time, and (3) even when NL abilities have been examined longitudinally, change in NL error signatures time has been inferred from statistical outcomes, rather than evaluated as part of a developmental model. The present study is designed to overcome these three limitations by examining NL error signatures on different NL tasks over time and by testing the viability of a LDS change model (McArdle, 2009; Coman et al., 2013).

In this study we use a LDS model to assess change and/or stability in NL error signatures over time (see Analytic Approach section for further model descriptions). Specifically, “change” in LDS models is defined as an explicit model parameter (i.e., the change score or latent difference), which is defined as the difference in scores between adjacent time points. When specified in this way, variance associated with the previous test occasion is removed from the change parameter. A benefit of this procedure is that “changes” between test occasions can be interpreted as independent of the accumulated changes from the initial start point (McArdle, 2009; Coman et al., 2013).

We examine children’s error signatures on 0–100 NL and 0–1000 NL tasks three times over an 18 month period. The change parameters in LDS models can be used to represent indices of cognitive change. If the change score derived from one NL task (e.g., change in estimation precision between T1 and T2 for 0–100 NL) predicts improvement in estimation on a different NL task (e.g., initial assessment of 0–1000 NL at T2 for instance), the statistical change parameters could be interpreted as capturing changes in an underlying (psychological) representational system^1^.

The linking of error signatures across NL tasks would suggest a set of common psychological indices independent of a particular number range. Conversely, if change in 0–100 NL scores predict less precise estimation on the 0–1000 NL task at T2, the change measure could be interpreted as reflecting experience or learning effects affecting a particular number range (in this case, the 0–100 NL) and not a change in the underlying representation. The failure to find a relationship in error signatures between a previously familiar number range (0–100 NL) and a less familiar number range (0–1000 NL) could be interpreted as suggesting an absence in an underlying representation.

If change in 0–100 NL error signatures across time predicts change in 0–1000 NL signatures across time (i.e., links between change parameters), it would suggest that the change processes are similar across task (i.e., the reduction in estimation in the 0–100 NL are similar to the reduction of errors in the 0–1000 NL task). On the other hand, an absence of a relationship would suggest that the rates of change differ. The latter effect would be unsurprising since improvements in 0–1000 NL abilities are likely to be still occurring.

In general, if a relationship between changes in NL error signatures and mental calculation were observed, it would suggest that improvement in NL estimation ability over time is related to math ability. It is possible that different change effects would be observed for the 0–100 NL and the 0–1000 NL, as well as the relationship between these effects and mental calculation ability (or any change in abilities). The latter information is important in determining whether NL error signatures per se are diagnostically useful in predicting math abilities.

## Materials and Methods

### Participants

The data were collected in sessions on four different occasions at approximately 6-monthly intervals over a 2 year period (hereafter referred as T1–T4). On the first occasion the mean age was 6.30 years (SD = 4.4 months). Participants comprised 217 children (59.5% male) and attended one of seven schools in middle-class suburbs of a large Australian city. All children spoke English, had normal or corrected to normal vision, and, according to school personnel, had no known learning disabilities. The study was conducted with the agreement of, and in compliance with, the requirements of the authors’ University’s Human Ethics Committee.

### Materials and Procedure

**FIGURE 1:**
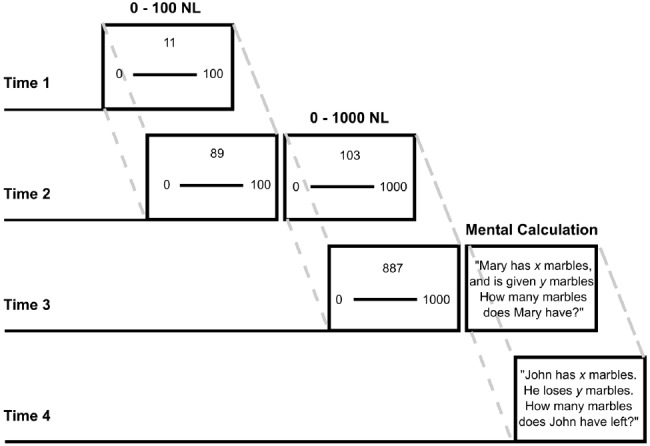
**Partially overlapping longitudinal design for Time 1—Time 4.** Example problems are shown for the 0–100 NL, 0–1000 NL, and mental calculation tasks.

Children completed Number Line Estimation tasks on three test occasions and they also completed a Mental Addition task on two occasions. A graphical representation of test sequences across occasions is reported in Figure 1. On the first occasion (T1) children completed a 0–100 NL task; on the second (T2) a 0–100 NL and a 0–1000 NL task; on the third (T3) occasion a 0–1000 NL task. On the third and fourth occasions (T3 and T4), children completed mental calculation tasks.

#### Number Line Estimation Task

A “number—position” task (e.g., Siegler and Opfer, 2003) was used on test occasions one to three (T1, T2, and T3). Children were presented with a sequence of A4 pages, one at a time, on which a 25 cm horizontal line was drawn. The left-hand end of each line was marked with a “0” and the right-hand marked with either “100” (0–100 NL) or “1000” (0–1000 NL). At the top center of each page a target number was printed in large bold type. Following Siegler and Opfer, children received three familiarization trials at the beginning of each test session. They were presented with one of the blank NL sheets and instructed to “note the number at the top of the page,” and use the pencil to mark “where that number belongs” on the line. Children were asked to indicate “where the number goes” as quickly as possible; however, we did not record “decision” times. All children appeared to grasp the aim of the task and marked numbers without hesitation.

Test trials immediately followed practice trials, prior to which task instructions were repeated. Children received no feedback on the accuracy of their responses. The 0–100 NL task comprised numbers “11, 29, 43, 61, 73, 89,” and the 0–1000 NL task numbers “103, 307, 401, 599, 701, 887.” Numbers were presented in a random order across individuals and sessions. We selected prime numbers as targets to limit the use of estimation strategies based on factorization. Children’s responses were analyzed using the average of absolute deviations from target numbers.

Analyses of NL error signatures often involve fitting algebraic functions to the magnitude of estimation errors of target numbers on a NL, which tends to show an age-related shift from a logarithmic to a linear fit function. Nevertheless, the shape of error functions should not be confused with the magnitude (fuzziness) of errors: NL error signatures can be linear but reflect relatively large imprecise (fuzzy) numerical representation (Moeller and Nuerk, 2011). Thus it is possible to use estimates that, when plotted against the actual magnitudes, fit perfectly a linear function that has neither the same slope or intercept value as the function for perfectly accurate responses (i.e., the function y = x). For this reason, absolute deviation scores may be a more useful index of NL performance since they are independent of model fit (see Ashcraft and Moore, 2012).

#### Mental Calculation

The mental calculation tasks comprised 24 arithmetic word problems, presented via the audio system of a laptop computer. The problems comprised eight joining problems (e.g., “Mary has x marbles. She is given y marbles. How many marbles does she have altogether?”), eight separating problems (e.g., “John has x marbles. He loses y marbles. How many marbles does he have left?”), and eight separating-joining problems (e.g., “John has x marbles. He lost y marbles. How many marbles did he have to begin with?”). The word problems are age appropriate and have been widely used previously to identify difference in math problem solving abilities (Carpenter and Moser, 1982; Butterworth, 2005). Prior to solving the 24 test problems, children completed three practice problems, one each of the three word problem types. They were instructed to listen very carefully to the number story problem and work out the answer as quickly as possible. If a child asked to hear a problem again, it was read again. To ensure children understood the task, they were asked to describe what the problems were asking them to do. We did not provide feedback on the accuracy of answers. The test phase commenced immediately following the practice session. Children’s answers were scored as correct or incorrect.

### Analytic Approach

**FIGURE 2:**
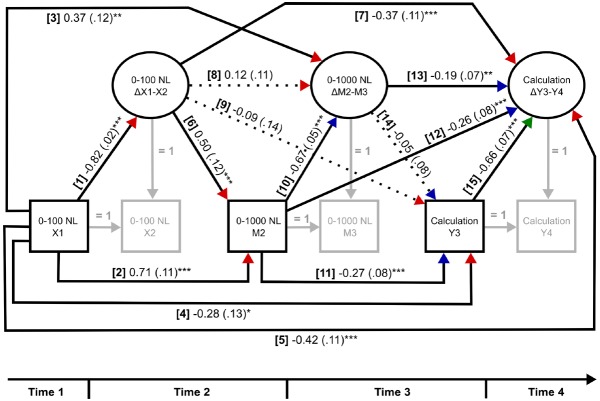
**Latent difference score mediation model with standardized direct effects (and standard errors).** Direct effects numbered [1]–[15] are interpreted in text. X1 = 0–100 NL predictor T1; X2 = 0–100 NL predictor T2; ΔX1–X2 = change in 0–100 NL predictor; M2 = 0–1000 NL mediator T2; M3 = 0–1000 NL mediator T3, ΔM2–M3 = change in 0–1000 NL mediator; Y3 = mental calculation outcome T3; Y4 = mental calculation outcome T4, ΔY3–Y4 = change in mental calculation outcome. Red arrow heads represent predictions relating to 0–100 NL, blue arrow heads with 0–1000 NL, and a green arrow head with mental calculation. *p < 0.05, **p < 0.01, ***p < 0.001.

We use a LDS model (McArdle, 2009; Selig and Preacher, 2009; Coman et al., 2013) to investigate stability/change in NL error signatures over time and task to determine if change parameters per se predict NL estimation accuracy and math problem solving over time. The “change” parameter in LDS models (labeled as Δ in the model, see Figure 2) corresponds to a latent/unobserved variable in the logic of structural equation modeling (SEM). These parameters of change (i.e., the change score or latent difference) are defined between adjacent time points for each variable of interest and are interpreted as capturing the uniqueness of a current measure that is separate to an immediate prior measure. When this method is repeated over time points and across tasks, LDS models include both the effects of prior performance (as with autoregressive effects in CLPMs), and importantly the effects of prior changes in performance. Specifying the model in this way allows an empirical test of inter-individual differences (between-person) as well as intra-individual changes (within-person) on the variable of interest. So-called “direct” and “indirect” effects can also be specified in this model and are interpreted in a similar manner to mediation analyses (Selig and Preacher, 2009). For instance, 0–100 NL accuracy at T1 may directly influence mental calculation abilities at T3 or this relationship may be indirectly expressed through 0–1000 NL accuracy at T2. Bias-corrected bootstrap confidence intervals are reported for all indirect effects based on 10,000 bootstrap samples, allowing for non-symmetric intervals.

Data for all test occasions were available for 186 of the 217 children (86%), with data from at least one occasion available for the remainder: missing T2 only (*n* = 1), missing T2–T3–T4 (*n* = 16), missing T3–T4 (*n* = 1), and missing T4 only (*n* = 13). Our model results and conclusions are not affected by whether these children are included in our analyses or not, and all missing data occurred for arbitrary reasons (e.g., child moved school, absence due to illness etc.). Full-information maximum likelihood estimation was used as a robust index of missing data. All model parameters were derived using robust maximum likelihood estimation in Mplus version 7 (Muthén and Muthén, 1998–2013).

## Results

### Descriptive Statistics

As expected, precision of NL errors improved for both the 0–100 NL and 0–1000 NL tasks over time. For 0–100 NL, average absolute deviations decreased between T1 (*M* = 2.04 cm, SD = 1.00 cm) and T2 (*M* = 1.42 cm, SD = 0.59 cm). For 0–1000 NL, average absolute deviations also decreased between T2 (*M* = 3.37 cm, SD = 1.98 cm) and T3 (*M* = 2.80 cm, SD = 1.78 cm). The proportion of mental calculation problem solved correctly improved over time (T3: *M* = 0.76, SD = 0.20; T4: *M* = 0.81, SD = 0.17—since we found no differences in the number of problems solved correctly as a function of problem type, the means represent proportion correct across 24 problems is reported). Paired sample *t*-tests confirmed that differences on the 0–100 NL [*t*(185) = –9.534, *p* < 0.001], 0–1000 NL [*t*(185) = –3.751, *p* < 0.001] and mental calculation [*t*(185) = 5.418, *p* < 0.001] tasks were all significant.

All tasks were correlated within and across test occasions (see Table 1). For both NL tasks, greater imprecision on the first test occasion was significantly associated with greater imprecision on the following test occasion. The same pattern of association was observed across NL tasks and test occasions. Greater imprecision for each NL task at T1–T3 was also significantly associated with lower mental calculation accuracy at both T3 and T4. Greater mental calculation accuracy at T3 was significantly associated with greater accuracy at T4.

**TABLE 1:** **Longitudinal correlations across T1–T4 for 0–100 NL, 0–1000 NL, and mental calculation tasks**.

****	**Time 1**	**Time 2**		**Time 3**		**Time 4**
	0–100NL^a^	0–100NL^a^	0–1000NL^a^	0–1000NL^a^	Calculation^b^	Calculation^b^
0–100NL^a^	1					
0–100NL^a^	0.37**	1				
0–1000NL^a^	0.30**	0.38**	1			
0–1000NL^a^	0.41**	0.32**	0.51**	1		
Calculation^b^	–0.29**	–0.22*	–0.32**	–0.25**	1	
Calculation^b^	–0.35**	–0.40**	–0.40**	–0.40**	0.68**	1

a, average absolute deviation; b, mental calculation total proportion correct. *p < 0.01, **p < 0.001.

Overall, the descriptive results are consistent with previous research which shows that NL estimation patterns are significantly associated across time and task, and that these NL estimation patterns are related to later math ability (see Landerl, 2013; LeFevre et al., 2013).

### Latent Difference Score Mediation Model

The LDS mediation model is shown in Figure 2, with standardized regression parameters printed along the associated longitudinal pathways. We report the direct effects first to determine whether NL error signatures persist over time and task and predict later mental calculation abilities. Indirect effects (shown in Table 2) are then interpreted to assess the possible existence of a mediated relationship between NL error signatures and later math performance. (As noted earlier, the interpretation of direct and indirect effects from the LDS model control for prior measures, as well as prior changes in these measures).

**TABLE 2:** **Indirect effects for the latent difference score mediation model**.

**No.**	**Predictor**	**Mediator**	**Outcome**	**Estimate**	**95% CI^a^**
[1]	0–100NL_*X*1_	→0–1000NL_*M*2_	→Calculation_*Y*3_	–0.192	[–0.324, –0.060]*
[2]	0–100NL_*X*1_	→Δ0–1000NL_*M*2–*M*3_	→Calculation_*Y*3_	–0.018	[–0.084, 0.048]
[3]	Δ0–100NL_X1-X2_	→0–1000NL_*M*2_	→Calculation_*Y*3_	–0.135	[–0.242, –0.029]*
[4]	Δ0–100NL_X1-X2_	→Δ0–1000NL_*M*2–*M*3_	→Calculation_*Y*3_	–0.006	[–0.035, 0.024]
[5]	0–100NL_*X*1_	→0–1000NL_*M*2_	→ΔCalculation_*Y*3–*Y*4_	–0.184	[–0.317, –0.050]*
[6]	0–100NL_*X*1_	→Δ0–1000NL_*M*2–*M*3_	→ΔCalculation_*Y*3–*Y*4_	–0.070	[–0.141, 0.001]
[7]	Δ0–100NL_X1-X2_	→0–1000NL_*M*2_	→ΔCalculation_*Y*3–*Y*4_	–0.129	[–0.235, –0.024]*
[8]	Δ0–100NL_X1-X2_	→Δ0–1000NL_*M*2–*M*3_	→ΔCalculation_*Y*3–*Y*4_	–0.022	[–0.066, 0.022]

Indirect effects numbered [1]–[8] are reported in text. X, predictor; M, mediator; Y, outcome; a, standardized bias-corrected bootstrap confidence intervals (10,000 samples); *confidence interval excludes 0 (i.e., significant indirect effect).

#### Direct Effects

Direct effects of error signatures on the 0–100 NL task persist over time and across tasks. Poorer estimation on the 0–100 NL task at T1 predicted [1] less change on the 0–100 NL task between T1 and T2, [2] poorer estimation on the 0–1000 task at T2, [3] greater change on the 0–1000 NL task between T2 and T3, [4] poorer mental calculation accuracy at T3, and [5] less change in calculation accuracy between T3 and T4. The link between earlier inaccurate NL estimation and poorer math outcomes (poorer mental calculation) replicates previous cross-sectional and longitudinal research.

Greater improvement in 0–100 NL estimation precision between T1 and T2 predicted, [6] relatively poorer estimation on the 0–1000 NL task at T2, and [7] less change in mental calculation accuracy between T3 and T4. This finding is unsurprising since it likely shows that greater improvement (from T1 to T2 on the 0–100 NL) is associated with initially poorer 0–1000 NL estimation errors. Improvement on the 0–100 NL task between T1 and T2 was [8] unrelated to similar improvements on the 0–1000 NL task between T2 and T3 (i.e., a so-called change-on-change effects) or [9] mental calculation accuracy at T3. The finding of unrelated changes across NL tasks suggests that such improvements reflect differences in change processes per se.

Error signatures on the 0–1000 NL task showed a similar pattern of predictive relationships to the 0–100 NL task. Poorer estimation on the 0–1000 NL task at T2 predicted, [10] less change on the 0–1000 NL task between T2 and T3, [11] poorer mental calculation accuracy at T3, and [12] less change in mental calculation accuracy between T3 and T4. Moreover, greater change on the 0–1000 NL task between T2 and T3 was related to [13] less change in mental calculation accuracy between T3 and T4, but did not predict [14] mental calculation accuracy at T3. The similarity of effects across both NL tasks strengthens the argument of persistence in NL error signatures over time, and ipso facto a similar underlying representation.

Lastly, mental calculation performance was relatively stable over time. Greater mental calculation accuracy at T3 predicted [15] less change in mental calculation accuracy between T3 and T4. Changes in accuracy were relatively small (0.76–0.81 problem correctly solved).

#### Indirect Effects

The eight indirect effects of the model, with associated non-symmetric 95% confidence intervals, are shown in Table 2. LDS model indirect effects are interpreted in a similar manner to mediation models; i.e., a unit increase in the predictor (“X”) predicts a change in the mediator (“M,” direct effect), which predicts a change in the outcome (“Y,” indirect effect).

All indirect paths with 0–1000 NL estimation at T2 as a mediator were significant. Conversely, all indirect pathways involving change in 0–1000 NL estimation between T2 and T3 as a mediator were non-significant. We interpret this to suggest that increases in proficiency 0–1000 NL may be still occurring and the change does not sufficiently mediate the relationship between the early NL error signatures and later mental calculation ability. The significant indirect effects are reported below (the Indirect effects are labeled 1, 3, 5, 7 in Table 2).

The persistence of poorer error signatures across NLs is related to poorer math outcomes. Poorer estimation for 0–100 NL at T1 predicted poorer estimation for 0–1000 NL at T2, “leading to” lower mental calculation accuracy at T3 (indirect effect 1). Greater change in 0–100 NL estimation between T1 and T2 predicted poorer estimation for 0–1000 NL at T2, leading to lower mental calculation accuracy at T3 (indirect effect 3). The fact that change in 0–100 NL is not related to better initial performance on the 0–1000 NL task suggests that this change reflects poorer initial 0–100 NL estimation ability.

A similar pattern of indirect effects were found when change in mental calculation accuracy was the predicted outcome. Poorer estimation for 0–100 NL at T1 predicted poorer estimation for 0–1000 NL at T2, leading to less change in mental calculation accuracy between T3 and T4 (indirect effect 5). Greater change in 0–100 NL estimation between T1 and T2 predicted poorer estimation for 0–1000 NL at T2, leading to less change in mental calculation accuracy between T3 and T4 (indirect effect 7).

## Discussion

A LDS model was employed to examine stability and changes parameters in NL error signatures over a 2 year period, and to examine the degree to which these changes are linked to mental calculation abilities. Four major findings are worth noting. First, the magnitudes of NL estimation error signatures for the 0–100 NL and the 0–1000 NL both declined over time and initial performance on both tasks were related; nevertheless, the rates of decline differed (i.e., are not linked). In particular, greater change occurred over the 0–100 range than the 0–1000 range over the same period of time but on different occasions. Also, change in 0–100 NL did not predict change in 0–1000 NL (e.g., Figure 2, direct effect 8). The pattern of findings shows that estimation abilities improved on both tasks, but not at a similar rate across the similar time-frames. This finding is not particularly surprising since it is likely that relative knowledge of 0–100 NL and 0–1000 NL differed in the age of the children studied. Nevertheless, the NL error signatures for the 0–100 NL and the 0–1000 NL were linked in terms of the relative magnitude of errors across time. This pattern of findings suggests that the acuity of the representation associated with NL error signatures is stable over time; in other words, supports stable NL representation.

Second, as expected from an analysis of the direct effects and subsequent paired-sample *t*-tests, the mean NL estimation errors on the 0–100 NL task were more precise at T2 compared to T1; and the estimation errors on the 0–1000 NL were more precise at T3 compared to T2. Nevertheless, the correlation between T1 and T2 for the 0–100 NL task and between T2 and T3 for the 0–1000 NL suggests that the relative error signatures remain relatively stable across time, which suggests a common representation system. Further, a similar pattern of relative error signatures occurred across tasks; error signatures on the 0–100 NL at T1 were related to the 0–1000 NL error signatures at T2. Similarly, the 0–100 NL at T2 was related to the 0–1000 NL error signatures at T3. These findings in particular suggest that NL error signatures remain relatively stable within and across NL task and across time. In other words, they suggest that the NL representation system remains relatively stable across time. In sum, while NL error signatures get smaller over time, the relative magnitude of the signatures remained. By itself these findings supports an interpretation that NL representations are relatively predictable over time. The latter interpretation however, is based on “direct effects” in the LDS model, and ignores the possible contribution of so-called “indirect effects” parameters in the model, which are considered below.

Third, mental calculation accuracy (and change in mental calculation accuracy from T3 to T4) could be predicted by NL error signatures, as well as changes in NL error signatures over time. In particular, less precise initial 0–100 NL and 0–1000 NL estimation predicted poorer mental calculation accuracy at T3 and less change in calculation accuracy between T3 and T4. It is also evident that mental calculation ability was relatively stable over time, with small incremental, correlated changes, found. And fuzzy NL error signatures were associated with poorer mental calculation abilities–a similar finding to that found in previous cross-sectional and longitudinal research (Schneider et al., 2009; Sasanguie et al., 2012b; Landerl, 2013). The fact that change in NL error signatures predicted less change in mental calculation ability may be partly attributed to accuracy remaining relatively high over time (76–81% of problems correct). Overall, it is evident that the LDS change parameters do indeed provide unique predictions for both NL error signatures and computation ability.

Fourth, the indirect pathway effects in the LDS model also reveal interesting effects. Of particular note, estimation error signatures on the 0–1000 NL at T2 mediate the 0–100 NL at T1 in predicting mental calculation at T3 (and the change in calculation between T3 and T4). This set of relationships is replicated for the T1 to T2 change in the 0–100 NL. Nevertheless, it should be noted that the T2 to T3 change in the 0–1000 NL did not similarly mediate this prediction. This finding suggests either that (1) insufficient change has occurred in the 0–1000 NL between T2 and T3, or (2) change in the 0–1000 NL is not associated with calculation ability per se. We suggest that the former interpretation is more likely to be the case.

However, we acknowledge that children may only need to improve their precision on the 0–100 NL task for a sufficient shift in numerical understanding to occur that is relevant for mental calculation. Improvements to 0–1000 NL estimation may be irrelevant in this case. Relatedly, the NL task may not be the most sensitive measure of mental magnitude representation for larger number ranges. Nevertheless, we believe these measurement issues are inherent in all studies designed to assess cognitive phenomena that change over time. Moreover, our findings correspond with both longitudinal (Gunderson et al., 2012; Landerl, 2013; LeFevre et al., 2013; Muldoon et al., 2013; Praet and Desoete, 2014) and experimental studies (Ramani and Siegler, 2008; Siegler and Ramani, 2008, 2009) of NL estimation. Consistent with previous research, our findings show that change in NL error signatures occurs relatively slowly over time (Siegler and Opfer, 2003; Siegler and Booth, 2004; Booth and Siegler, 2006, 2008; Thompson and Opfer, 2010; White and Szucs, 2012).

### Conclusion

Overall, the findings show a relative consistency in NL representations over time and task, as indexed by the NL estimation error signatures. Moreover, specific changes in NL representations per se are important predictors of concurrent and future arithmetic problem solving. It is evident that the LDS model framework provides information about change not revealed by other longitudinal analytic approaches (e.g., longitudinal regression analyses, cross-lagged panel models). In our view, LDS models overcome some of the limitations of earlier longitudinal research which have used different analytic models. Although our findings are consistent with earlier longitudinal NL findings, they provide a more solid basis for drawing inferences about the relationships between changes in NL representations and calculation abilities.

Here we note three points. First, our research approach goes beyond comparing differences in changes in NL error signatures (Landerl, 2013) to making predictions about the stability and change in error signatures within and between NL error signatures across time and their relationship to computation abilities. In other regression approaches, change is inferred from the significance of longitudinal “pathways” in the model; in LDS, in contrast, change is an explicit parameter in the model. Second, we were able to make predictions about stability and change, rather than simply focusing on the autoregressive effects associated with stability models (LeFevre et al., 2013). It is evident that in the LDS approach, we were able to tease apart the differential impact of change and stability factors. Third, even where researchers have attempted to fit latent growth models (Muldoon et al., 2013), methodological limitations have impeded full model convergence occurring. In LDS models change is specified in terms of adjacent time points, and is thus less restrictive than the requirements associated with fitting a curve in latent growth models, as used by Muldoon et al. (2013), and Praet and Desoete (2014) in their analyses of NL change. Our pattern of findings suggests that modeling an entire relational change network provided a convergent model. Overall, the findings support the claim that a common representation NL error signature system underlies estimate judgments and that this system is associated with computation accuracy.

### Conflict of Interest Statement

The authors declare that the research was conducted in the absence of any commercial or financial relationships that could be construed as a potential conflict of interest.

## Footnotes

1 Note: “stability” and “change” have both statistical and psychological meanings. Statistically, “stability” tends to refer to orderly relationships among measures, which may nevertheless change in a lawful ways over time. Psychologically, “stability” tends to be regarded as a persistence of relationships that do not change (over time). Here, we use the term “stability” as a statistical referent, and “persistence” as a psychological referent. The statistical and psychological meanings of “change” however, are very similar. Because of the similarity in meaning, we used “change” interchangeably. The significance of stability/change parameters for a developmental account of NL error signatures are critical; and in particular, specifying the developmental trajectories that might predict math ability.
